# Duration and frequency of migraines affect cognitive function: evidence from neuropsychological tests and event-related potentials

**DOI:** 10.1186/s10194-017-0758-6

**Published:** 2017-05-05

**Authors:** Lifang Huang, Hong juan Dong, Xi Wang, Yan Wang, Zheman Xiao

**Affiliations:** Department of Neurology, Renmin Hospital of Wuhan University, Wuhan, 430060 Hubei Province People’s Republic of China

**Keywords:** Migraine, Duration, Frequency, Cognitive deficits, Event-related potentials

## Abstract

**Background:**

The aim of this study was to evaluate the changes in the cognitive performance of migraine patients using a comprehensive series of cognitive/behavioral and electrophysiological tests.

**Method:**

A randomized, cross-sectional, within subject approach was used to compare neuropsychological and electrophysiological evaluations from migrane-affected and healthy subjects.

**Results:**

Thirty-four patients with migraine (6 males, 28 females, average 36 years old) were included. Migraineurs performed worse in the majority of the Montreal Cognitive Assessment (MoCA) (*p* = 0.007) compared to the healthy subjects, significantly in language (*p* = 0.005), memory (*p* = 0.006), executive functions (*p* = 0.042), calculation (*p* = 0.018) and orientation (*p* = 0.012). Migraineurs had a lower score on the memory trial of the Rey–Osterrieth complex figure test (ROCF) (*p* = 0.012). The P3 latency in Fz, Cz, Pz was prolonged in migraineurs compared with the normal control group (*P* < 0.001). In addition, we analyzed significant correlations between MoCA score and the duration of migraine. We also observed that a decrease in the MoCA-executive functions and calculation score and in the ROCF-recall score were both correlated to the frequency of migraine. Migraineurs were more anxious than healthy subjects (*p* = 0.001), which is independent of cognitive testing. Differences were unrelated to age, gender and literacy.

**Conclusions:**

Cognitive performance decreases during migraine, and cognitive dysfunction can be related to the duration and frequency of a migraine attack.

## Background

Migraine is the second most prevalent type of primary headache disorder, which affects approximately 14.7% of both males and females [1]. Migraine is characterized by moderate to severe throbbing pain with sensitivity or intolerance to light and sound during the headache and is often accompanied by nausea and vomiting. Migraine, especially chronic migraine, is often comorbid with psychiatric disorders that exhibit affective temperament dysregulation and suicidal behaviors [2, 3]. Migraine has been associated with an increased risk of vascular events, specifically cardiovascular disease [4]. Vascular pathology is a strong risk factor for cognitive dysfunction [5, 6].

In addition, migraine has been linked to an increased prevalence of clinically silent brain lesions and subtle gray matter damage which affect the cognitive processes [7, 8]. These associations suggest that individuals with migraines have impaired cognitive function due to these structural lesions. However, previous studies of migraine and cognition have been contradictory. Some studies suggest that migraineurs have subtle interictal cognitive abnormalities aligning with attentional deficits [9–11]. Some studies have reported improved performance [12]. Some longitudinal studies have suggested fewer dysfunctions in the Mini Mental State Examination (MMSE) and executive functions in migraineurs. In addition, some studies have not found any significant differences in cognitive performance between migraine patients and controls [13]. These inconsistencies could be due to methodological issues including different migraine assessment methods and the program of cognitive assessment.

Given the high prevalence of migraine in the general population, it is clear that the relationship between cognitive dysfunction and migraines present a significant public health interest.

Event-related potentials (ERPs) are one of the most useful tools in investigating neural substrates and cerebral regions involved in specific cognitive function because of the objectivity and noninvasiveness of the method. Undoubtedly, the P3 is the most studied cerebral wave used for evaluating cerebral information processing during the course of various neurological diseases because of its easy recording and reliability.

This study aimed to characterize cognitive testing and P3 in migraineurs. We hypothesized that migraine patients have cognitive dysfunction and P3 abnormalities, such as reduced P3 amplitude and/or a prolonged latency, suggesting alterations in the cognitive-evaluative component.

## Methods

### Participants

We recruited 34 migraine patients (6 males, 28 females; mean age 36 years; range between 20 to 55 years) from the Renmin Hospital of Wuhan University, 10 with aura and 24 without aura. Patients were not receiving prophylactic therapy, were medicine-free for at least 24 h and were in the interictal period when recruited. We also recruited 24 healthy age-matched participants (6 males, 18 females; mean age 36 years; range between 22 to 58 years) with no history of headache or drug/alcohol abuse. Patients and controls had normal or corrected-to-normal vision. We excluded participants who were illiterate or suffering from depression, stroke, or brain injuries. All participants provided written and informed consent prior to commencement of the experiment.

### Assessment of migraines

Participants were diagnosed based on diagnostic criteria of the International Classification of Headache Disorders (ICHD-3 beta).

### Evaluation of cognitive functions

The Montreal Cognitive Assessment (MoCA) was included as a screening test of general cognitive abilities. Several separate domains of cognitive function were covered including visuospatial functions, attention, language, memory, executive functioning, calculation and orientation. It took approximately 10 min to administer the MoCA. The maximum possible score was 30 points, and a score of 26 or above was considered normal. The Rey–Osterrieth Complex Figure Test (ROCF) was used to assess visual perception, constructional praxis, and visuo-spatial memory, and was adapted from Osterrieth [14]. First, subjects were given the ROCF stimulus card, and they were asked to draw the same figure. Then, after a delay of 20 min, they were required to draw what they remembered. Study variables were as follows: (a) copy accuracy: quality of the copy as a reflection of visuo-constructive ability; (b) delayed recall: accuracy of the figure after the 20 min delay. According to the scoring system, the figure contained 18 elements, each of which received a score of 0.5, 1 or 2, depending on the accuracy, deformation, and location of each element. The full score was 36 for each of the 2 tasks. Basic information processing speed was measured with the Digit Symbol Substitution Test (DSST). In this test, the time needed to copy symbols in boxes that were indexed by digits, according to a key of digit/symbol combinations at the top of a sheet of paper. Under each digit, the corresponding symbol should be written as fast as possible. The time for completion of all symbols was measured. A history of depression and anxiety was determined based on the responses of the participants to a series of questions used to diagnose these mental disorders, called the Self-Rating Anxiety Scale and Self-Rating Depression Scale.

### Electrophysiological study

#### Procedure

The experiment was performed in a sound-attenuated room with a dim light. Participants were seated in a comfortable armchair and were presented with a white fixation cross at the center of a 19 in computer monitor placed 50 cm in front of them. Two hundred stimuli were presented in a random fashion. Stimuli were comprised of target stimuli (the number “2”, 20% probability of presentation) and standard stimuli (the number “8”, 80% probability of presentation). The duration of each stimulus was 50 ms, and the inter-stimulus interval was randomly varied between 800 ms and 1200 ms. All the stimuli were generated using the E-prime software. Participants were instructed to press the button as quickly and accurately as possible when they viewed the target stimuli. Accuracy was defined as the rate of pressing the button when viewed the target stimuli. Reaction time was defined as the length between viewing the target stimuli and pressing the button accurately. The accuracy and reaction time recordings were accomplished using E-prime software 2.0.

#### ERP recording and measurement

The electroencephalogram (EEG) was continuously recorded at Fz, Cz and Pz electrode sites according to the 10–20 International System with 36 Ag/AgCl active electrodes via NuAmps EEG/ERPs 36 Channel Amplifier of NEURO SCAN LABS. The electrode site on the scalp was prepared with alcohol and scraped with scrub cream to remove cutin resulting in an electrode impedance of less than 10 kΩ. Electrode impedance was kept below 10 kΩ throughout the experiment. All scalp electrodes were referenced to the average of the left and right mastoid signals. To ensure proper eye fixation and to allow for the removal of events associated with eye movement artifacts, vertical and horizontal eye movements were recorded with two pairs of electrodes, one placed above and below the left eye, and the other 10 mm from the lateral canthus of both eyes. High-pass and low-pass filters were set at 1.0 Hz and 30.0 Hz, respectively. The sampling rate was 1000 Hz.

For each participant, the EEG was segmented into the epoch from 200 ms pre-stimulus to 800 ms post-stimulus via Curry7.0 software. These single-subject waveforms were then used to generate the group-averaged waveforms for display and analysis. A -200 to 0 ms pre-stimulus baseline was used for all ERP waveform measurement and displays. Trials with signal exceeding ±200 mV amplitude in any recording channel were excluded from averaging. The EEG segments were averaged separately for target and standard stimuli. Only correctly detected responses were averaged. The number of averaged trials left after removal of the artifacts was 40 (target) and 160 (standard).

The difference waveform was obtained by subtracting ERPs in response to standard stimuli from those in response to target stimuli. The positive peak between 300 and 500 ms were used to define the P3 component. The peak amplitudes and latencies for the P3 component were measured relative to the pre-stimulus baseline period.

### Statistical analysis

We first used the Shapiro-Wilk test to assess the distribution of the variables. Quantitative data were presented as the mean ± standard deviation or median. Student’s *t*-test for independent samples (two-tailed) was used to compare the two groups in the scores of cognitive test, p3 amplitude and latency. Correlations between clinical (illness duration and frequency of attacks), cognitive test (MoCA, ROCF, DSST) and electrophysiological (ERP components amplitude and latency) data were computed. Pearson product-moment correlation coefficients (r) were reported. Statistical calculations were carried out using SPSS20.0. All the results were considered to be significant at *p* < 0.05.

## Results

### Sample characteristics

Table 1 shows demographic and clinical characteristics of migraine. There was no statistically significant difference between migraineurs and healthy subjects in age and education level.

**Table 1:** Sample Characteristics

	Migraineurs	Healthy subjects	*P*
	Mean ± SD	Mean ± SD	
*N*	34	24	
Age, years	36.065 ± 10.046	36.052 ± 12.968	0.997
Education, years	13.032 ± 4.231	13.790 ± 5.298	0.580
Headache score,	6.383 ± 1.670	–	–
Headache frequency, per month	3.532 ± 3.956	– –	– –
Duration of headache, hours	23.4 ± 24.197	–	–
Headache history, years	11.25 ± 9.290	–	–
SCL anxiety score	33.667 ± 8.671	26.737 ± 3.769	0.001*
SCL depression score	33.000 ± 9.390	28.368 ± 5.479	0.064

*SCL* symptom checklist, *P < .05

In our study, migraineurs suffered 3.532 (SD 3.956) headaches a month, with the average headache lasting 23.4 h (SD 24.197). On average, migraine participants had been having migraines for 11 years.

Emotional characteristics of both groups were compared: the migraine group was significantly more anxious than the healthy groups.

### Comparisons of cognitive testing

Mean scores on the MoCA, digit symbol substitution test and Rey–Osterrieth complex figure test are shown in Table 2. Significant differences were observed in the MoCA (*p* = 0.007), especially in language, memory, executive functions, calculation and orientation. Migraineurs had a lower score on the memory trial of Rey–Osterrieth complex figure test compared to healthy controls (*p* = 0.012). Performance on the digit symbol substitution test and copy trial of Rey–Osterrieth complex figure test was not significantly different.

**Table 2:** The Cognitive Outcome measures between migraine group and control group

	Migraineurs	Healthy subjects	F	*P*
	Mean ± SD	Mean ± SD		
MoCA	25.677 ± 4.291	28.048 ± 1.829	7.664	0.007*
Visuospatial function	3.618 ± 0.604	3.550 ± 0.999	2.252	0.817
Attention	2.706 ± 0.676	2.850 ± 0.366	5.218	0.248
Language	4.971 ± 1.243	5.714 ± 0.643	5.687	0.005*
Executive functions	6.824 ± 1.714	7.600 ± 0.940	6.717	0.042*
Calculation	2.735 ± 0.618	3.000 ± 0.000	19.349	0.018*
Memory	4.029 ± 1.507	5.096 ± 0.995	3.677	0.006**
Orientation	5.824 ± 0.387	6.000 ± 0.00	25.357	0.012*
DSST’s	122.917 ± 46.404	106.684 ± 36.002	0.090	0.217
ROCF-copy	35.792 ± 0.833	35.684 ± 0.946	0.684	0.694
ROCF-recall	20.688 ± 6.129	25.316 ± 5.218	0.308	0.012*

Visuospatial function refers to the copy cube and draw a clock test; Attention refers to the vigilance test and digit span test; Language function refers to naming, sentence repetition and verbal fluency test; Executive functions refers to the alternating trail making, abstraction, verbal fluency, copy cube and draw a clock test; Calculation refers to serial 7 s test; Memory contains delayed recall and verbal fluency test; *P < .05, **P < 0.001

### ERP data

There was no significant group difference in accuracy (migraineurs, 97.41%; healthy subjects, 98.38%; *P* = 0.187), or in the appropriate reaction time between migraineurs (449.1 ms) and healthy subjects (445.1 ms; *P* = 0.272).

Table 3 shows the results of P3 amplitude and latency between the migraine group and healthy group. The latency was longer in the migraine group compared to the healthy subjects (*P* < 0.001). In the migraine group, the P3 amplitude varied between sites, was maximal in the parietal lobe (Pz: 9.906 μV) and minimal in the frontal lobe (Fz: 8.243 μV), with intermediate values at Cz (8.735 μV), but the difference did not reach statistical significance (*P* = 0.319). There was no significant difference between the location of recording: left (Pz3) vs. right (Pz4) vs. midline (Pz).

**Table 3:** Results of P3 amplitude and latency between migraine group and control group

ERP		Migraineurs	Healthy subjects	F	*P*
P3		Mean ± SD	Mean ± SD		
Amplitude (μV)	Fz	8.243 ± 3.964	9.318 ± 3.106	2.474	0.285
	Cz	8.735 ± 4.262	8.973 ± 3.055	1.515	0.820
	Pz	9.906 ± 4586	10.859 ± 3.191	3.337	0.394
Latency (ms)	Fz	406.517 ± 37.552	368.417 ± 12.552	16.253	0.000**
	Cz	406.207 ± 38.097	366.833 ± 17.189	10.775	0.000**
	Pz	408.724 ± 36.748	367.792 ± 17.290	7.814	0.000**

*P < .05, **P < 0.001

### Correlations between cognitive scores and headache characteristics

We observed that the MoCA score showed a significant, negative and moderate correlation with the duration of migraine, especially in the aspects of language, executive functions, calculation and memory. In addition, the time for the digit symbol substitution test showed a positive correlation with duration of headache. We also observed a significant relationship between MoCA-executive functions, calculation score, ROCF-recall score and the frequency of migraine, whereas the remaining cognitive measures and the results of ERP did not correlate with headache characteristics (Table 4).

**Table 4:** Correlation between cognitive scores and headache characteristics in migraine patients

	Migraine score	Migraine frequency	Duration of migraine	Migraine history	SDS	SAS
Cognitive domains	r (*p* value)	r (*p* value)	r (*p* value)	r (*p* value)	r (*p* value)	r (*p* value)
MoCA	−0.194(0.270)	−0.311(0.073)	−0.478(0.004)**	−0.093(0.601)	−0.154(0.454)	−0.193(0.345)
Visuospatial functions	−0.030(0.865)	−0.290(0.096)	−0.257(0.142)	−0.201(0.255)	−0.296(0.142)	−0.315(0.117)
Attention	−0.054(0.762)	−0.143(0.420)	−0.186(0.293)	−0.162(0.361)	−0.283(0.161)	−0.208(0.308)
Language	−0.257(0.142)	−0.221(0.209)	−0.430(0.011)*	−0.149(0.401)	−0.195(0.339)	−0.232(0.255)
Executive functions	−0.165(0.351)	−0.458(0.006)**	−0.405(0.018)*	−0.037(0.835)	−0.228(0.262)	−0.225(0.268)
Calculation	−0.148(0.405)	−0.350(0.042)*	−0.446(0.008)**	−0.221(0.209)	−0.319(0.112)	−0.304(0.132)
Memory	−0.073(0.683)	0.042(0.815)	−0.374(0.030)*	0.058(0.746)	0.1490.468)	0.007(0.974)
Orientation	0.259(0.139)	−0.081(0.649)	−0.115(0.516)	0.059(0.741)	0.178(0.383)	0.238(0.243)
DSST	0.300(0.107)	0.132(0.486)	0.446(0.014)*	−0.175(0.355)	0.207(0.343)	0.270(0.212)
ROCF-recall	−0.318(0.087)	−0.415(0.023)*	−0.078(0.683)	0.075(0.692)	−0.207(0.344)	−0.336(0.117)
ERP Amplitude	−0.173(0.371)	0.136(0.371)	−0.029(0.881)	0.000(0.998)	−0.382(0.072)	−0457(0.028)*
ERP Latency	0.017(0.931)	−0.070(0.718)	−0.100(0.606)	0.093(0.630)	0.316(0.141)	0.312(0.147)

*P < .05, **P < 0.001

## Discussion

The present study revealed that migraine patients had significantly lower scores than controls on cognitive tests and increased P3 latencies. Our findings suggest that migraineurs are associated with cognitive dysfunctions. Previously, Freitas and colleagues found that 49% of MoCA score variability was attributed to age and education [15]. To exclude the influence of age and education on cognitive function, we had to balance age and education level between migraineurs and healthy subjects when we collected subjects. All participant ages were below 55 years old, and all had at least five years of primary education. For the psychological profile, migraineurs reported higher scores on the symptom checklist for anxiety. Some studies have shown that even subclinical levels of anxiety may negatively impact cognitive ability [16, 17]. To eliminate the effect of psychology, Spearman’s correlations were calculated between cognitive tests and psychology tests, and we did not find any correlation between cognitive tests and severity of psychological impairment. The poorer cognitive performance exhibited by migraineurs was not determined by the emotional variables.

The present study revealed that migraineurs had significantly lower scores than healthy subjects on the delayed recall of ROCF test, the total MoCA and in five out of seven cognitive subdomains (i.e., language, executive functions, calculation, memory and orientation domains). The MoCA was included as a screening test for general cognitive abilities and out of a maximum score of 30 points, the cut-off value was 26 points [18]. Eight migraineurs achieved scores below the available cut-off values in the MoCA test, but all of the scores were within two standard deviations of the mean. Four of them had low education, and two were elder than 50 years old. Most of the participants’ scores were not lower than the cut-off value reported in normative studies. Thus, we deemed that the reduced efficiency in selected cognitive domains did not correspond to a clinically relevant cognitive deterioration.

The longer durations and the increased frequency of migraine attacks were correlated with decreased cognitive performance. This indicates that the reduced scores on cognitive tests observed in migraineurs compared with controls is directly related to the length of time and the frequency of patient’s suffering from migraine.

Gil-Gouveia R aimed to document changes in cognitive performance of migraineurs under two conditions-during a naturally occurring untreated migraine attack and during a headache-free period. It was found that cognitive performance decreased during migraine attacks, especially in reading and processing speed, verbal memory and learning, supporting patients’ subjective complaints. Differences were found unrelated to pain intensity or duration of the attack [19]. Riva D assessed the effect of migraine on the interictal functioning of children and adolescents and observed that both patient groups had cognitive performance within a normal range, except for a significant delay in the reaction time task. These researchers considered that slower reaction time to simple visual stimuli may be an early sign of a subclinical neuropsychological dysfunction, significantly correlated with the frequency of headache attacks [20]. These data, together with our findings, would favor the view of some neuropsychological abnormalities in migraineurs as a consequence of the disease, or a perpetuating factor.

P3 is a generic name for a variety of relatively late, positive components with a centro-parietal midline distribution. Two principal electrophysiological markers have been considered as an objective index of cognitive processing: latency and amplitude. It is widely accepted that P3 latency reflects the length of stimulus evaluation processes [21], when two choice reaction time (RT) is required and its amplitude is largely determined by stimulus relevance, the amount of attention allocated to the stimulus, working memory and the task’s complexity [22, 23].

In our study, the latency for ERP components was significantly increased in migraine subjects. According to the previously mentioned facts, it could be suggested that migraineurs have a prolonged latency of P3, which represents a prolonged cognitive processing time. Some studies have shown that the amplitudes of P3 were significantly decreased in migraine patients compared with the healthy subjects, but the latencies of P3 did not show any significant effects [24, 25]. Other results show significant elongation of latencies and a dysfunction in P3 amplitudes in the migrant group during headache attacks [26]. However, several results have shown that migraine sufferers had longer P3 latencies, which is consistent with our findings.

The divergence among other studies and ours might be due to the different patient selection procedures. In our study, migraine patients were identified by expert neurologists according to established diagnostic criteria of the International Classification of Headache Disorders (ICHD-3 beta), while in previous studies, migraine patients were selected from a cohort of subjects on the basis of self-report measures. This procedure might have led to a misclassification of the non-migraine subjects. Furthermore, some articles have studied the cognition of children and adolescents. Cognition in children is immature, and we cannot compare it with adult cognitive function. Another potentially important factor is the procedure used for ERP. Differences in the tasks may probe different cognitive domains, and may lead to different conclusions.

In our study, ERP latency did not correlate with headache characteristics. This finding is not consistent with our expectations. ERP latency is a sensitive index, which is affected by many factors. The prolongation of latency may be affected by the migraine itself, but not by any characteristic of the headache.

In summary, the electrophysiological and behavioral data suggest that migraine is associated with cognitive dysfunctions, particularly, that cognitive dysfunctions can be related to the duration and the frequency of headache. Previously, people paid more attention to the pain of migraines and usually ignored the impairments of cognitive function in migraineurs. Although this functional phenomenon is present in the absence of clinically relevant deficits, it may reflect a vulnerability to executive high-demanding conditions of daily living activities in patients with migraine.

The strengths of this study include its standardized assessment of migraine status, information on migraine characteristics, and the availability of validated cognitive function measures. We discussed the correlations between neuropsychology, electrophysiology and migraine characteristics. We ruled out the effect of emotion on cognition. Our study had several limitations when interpreting our results. The study sample limited the ability to examine the effects of gender, age and education. Additionally, a previous study has shown that migraineurs with aura tend to exhibit even less decline over time than migraineurs overall [27]. We did not find differences in mean cognitive scores between migraineurs with and without aura. However, this was likely to due to the sample sizes and resulting low statistical power. De Tommaso and colleges found that the changes in brain responsivity are associated with various stages of the migraine cycle, since migraine patients seem to have a response augmentation and poor habituation that normalizes just before/during attacks [28]. In our study, patients were observed outside migraine attacks, but they could be at different points of a migraine cycle. We did not record the last time that they had a migraine attack. Furthermore, we did not observe the cognition of migraine patients at multiple time points, which limited us from examining the change in cognitive function over time.

## Conclusions

Cognitive performance decreases during migraine, especially in language, memory, executive functions, calculation and orientation, and cognitive dysfunctions can be related to the duration and the frequency of headache. These findings suggest the existence of brain dysfunction during attacks of migraine, which can relate specifically to migraine. We believe that it is necessary to shorten the duration and reduce the frequency of migraine attack. Careful clinical evaluation should be performed in migraine patients in order to diagnose early cognitive dysfunction and, if available, have them treated properly.

## References

[1] Bigal ME, Lipton RB (2009). The epidemiology, burden, and comorbidities of migraine. Neurol Clin.

[2] Serafini G, Pompili M, Innamorati M, Gentile G, Borro M, Lamis DA, Lala N, Negro A, Simmaco M, Girardi P, Martelletti P (2012). Gene variants with suicidal risk in a sample of subjects with chronic migraine and affective temperamental dysregulation. Eur Rev Med Pharmacol Sci.

[3] Pompili M, Di Cosimo D, Innamorati M, Lester D, Tatarelli R, Martelletti P (2009). Psychiatric comorbidity in patients with chronic daily headache and migraine: a selective overview including personality traits and suicide risk. J Headache Pain.

[4] Vetvik KG, MacGregor EA (2017). Sex differences in the epidemiology, clinical features, and pathophysiology of migraine. Lancet Neurol.

[5] Vermeer SE, Prins ND, den Heijer T, Hofman A, Koudstaal PJ, Breteler MM (2003). Silent brain infarcts and the risk of dementia and cognitive decline. N Engl J Med.

[6] Leys D, Henon H, Mackowiak-Cordoliani MA, Pasquier F (2005). Poststroke dementia. Lancet Neurol.

[7] Schwedt TJ, Dodick DW (2009). Advanced neuroimaging of migraine. Lancet Neurol.

[8] de Groot JC, de Leeuw FE, Oudkerk M, van Gijn J, Hofman A, Jolles J, Breteler MM (2000). Cerebral white matter lesions and cognitive function: the Rotterdam scan study. Ann Neurol.

[9] Demarquay G, Caclin A, Brudon F, Fischer C, Morlet D (2011). Exacerbated attention orienting to auditory stimulation in migraine patients. Clin Neurophysiol.

[10] Mickleborough MJ, Truong G, Handy TC (2011). Top-down control of visual cortex in migraine populations. Neuropsychologia.

[11] Mickleborough MJ, Hayward J, Chapman C, Chung J, Handy TC (2011). Reflexive attentional orienting in migraineurs: the behavioral implications of hyperexcitable visual cortex. Cephalalgia.

[12] Wen K, Nguyen NT, Hofman A, Ikram MA, Franco OH (2016). Migraine is associated with better cognition in the middle-aged and elderly: the Rotterdam study. Eur J Neurol.

[13] Martins IP, Gil-Gouveia R, Silva C, Maruta C, Oliveira AG (2012). Migraine, headaches, and cognition. Headache.

[14] Shin MS, Park SY, Park SR, Seol SH, Kwon JS (2006). Clinical and empirical applications of the Rey-Osterrieth complex figure test. Nat Protoc.

[15] Freitas S, Simoes MR, Alves L, Santana I (2012). Montreal cognitive assessment: influence of sociodemographic and health variables. Arch Clin Neuropsychol.

[16] Stillman AN, Rowe KC, Arndt S, Moser DJ (2012). Anxious symptoms and cognitive function in non-demented older adults: an inverse relationship. Int J Geriatr Psychiatry.

[17] Wetherell JL, Reynolds CA, Gatz M, Pedersen NL (2002). Anxiety, cognitive performance, and cognitive decline in normal aging. J Gerontol B Psychol Sci Soc Sci.

[18] Nasreddine ZS, Phillips NA, Bedirian V, Charbonneau S, Whitehead V, Collin I, Cummings JL, Chertkow H (2005). The montreal cognitive assessment, MoCA: a brief screening tool for mild cognitive impairment. J Am Geriatr Soc.

[19] Gil-Gouveia R, Oliveira AG, Martins IP (2015). Cognitive dysfunction during migraine attacks: a study on migraine without aura. Cephalalgia.

[20] Riva D, Aggio F, Vago C, Nichelli F, Andreucci E, Paruta N, D’Arrigo S, Pantaleoni C, Bulgheroni S (2006). Cognitive and behavioural effects of migraine in childhood and adolescence. Cephalalgia.

[21] McCarthy G, Donchin E (1981). A metric for thought: a comparison of P300 latency and reaction time. Science.

[22] Wang R, Dong Z, Chen X, Liu R, Zhang M, Wu J, Yu S (2014). Cognitive processing of cluster headache patients: evidence from event-related potentials. J Headache Pain.

[23] Kok A (2001). On the utility of P3 amplitude as a measure of processing capacity. Psychophysiology.

[24] Chen W, Shen X, Liu X, Luo B, Liu Y, Yu R, Sun G, Shen M, Wang W (2007). Passive paradigm single-tone elicited ERPs in tension-type headaches and migraine. Cephalalgia.

[25] Wang R, Dong Z, Chen X, Zhang M, Yang F, Zhang X, Jia W, Yu S (2014). Gender differences of cognitive function in migraine patients: Evidence from event-related potentials using the oddball paradigm. J Headache Pain.

[26] Titlic M, Mise NI, Pintaric I, Rogosic V, Vanjaka-Rogosic L, Mihalj M, Jurinovic P, Katic AC, Andjelinovic M (2015). The event-related potential p300 in patients with migraine. Acta Inform Med.

[27] Kalaydjian A, Zandi PP, Swartz KL, Eaton WW, Lyketsos C (2007). How migraines impact cognitive function: findings from the Baltimore ECA. Neurology.

[28] de Tommaso M, Ambrosini A, Brighina F, Coppola G, Perrotta A, Pierelli F, Sandrini G, Valeriani M, Marinazzo D, Stramaglia S, Schoenen J (2014). Altered processing of sensory stimuli in patients with migraine. Nat Rev Neurol.

